# The relative effect of mammographic screening on breast cancer mortality by socioeconomic status

**DOI:** 10.1097/MD.0000000000004335

**Published:** 2016-08-07

**Authors:** Theodora M. Ripping, Danielle van der Waal, André L.M. Verbeek, Mireille J.M. Broeders

**Affiliations:** a Radboud Institute for Health Sciences, Radboud University Medical Center; b Dutch Reference Center for Screening, Nijmegen, the Netherlands.

**Keywords:** breast cancer, case-control study, mammographic screening, mortality, socioeconomic status

## Abstract

Breast cancer incidence and mortality are higher in women with a high socioeconomic status (SES). The potential to prevent death from breast cancer is therefore greater in the high SES group. This does, however, require that the effectiveness of screening in the high SES group is equal to or greater than the effectiveness in the low SES group. The aim of this study is to assess the relative effectiveness of mammographic screening on breast cancer mortality by SES.

In Nijmegen, the Netherlands, women are invited to participate in biennial mammographic screening since 1975. Postal code is collected at each round and is used to calculate the SES of each woman based on the SES indicator of the Netherlands Institute for Social Research. The Dutch average was used to classify the SES score of each woman as either high or low. We designed a case-control study to investigate the effect of mammographic screening in women aged 50 to 75, 40 to 75, and 50 to 69 years, and calculated the odds ratios (ORs) and 95% confidence intervals (CIs).

Among the women invited to the mammographic screening program in Nijmegen, 10% had a high SES. In women aged 50 to 75 years, the breast cancer death rate was 38% lower in screened women than in unscreened women. The ORs for women with high SES (OR 0.82, 95% CI 0.31–2.19) and low SES did not differ significantly (OR 0.61, 95% CI 0.47–0.78).

Mammographic screening reduces breast cancer mortality, but we did not observe a significant difference in the relative effectiveness of screening by SES. If the effectiveness of mammographic screening is indeed not dependent on SES status, the absolute number of breast cancer deaths prevented by mammographic screening will be greater in the high SES than low SES group, because women with a high SES have a greater risk of breast cancer death.

## Abstract 2

Supplemental Digital Content is available in the text

## 1 Introduction

Many European countries have implemented a population-based mammographic screening program.^[1]^ Virtually all programs define the target population invited to screening by age alone. Because mammographic screening benefits some women and harms others,^[2,3]^ the question is arising whether mammographic screening can be optimized, that is, obtaining a more favorable benefit–harm balance. A more favorable balance between the benefits and harms of screening may be achieved by more targeted screening, that is, defining a target population for screening based on more risk factors in addition to age.

A potential risk factor for breast cancer that can be considered in this respect is socioeconomic status (SES). SES is associated with a range of risk factors for breast cancer, such as a mother's age at first childbirth and alcohol consumption.^[4–6]^ In addition, previous research has shown that women with higher SES have a higher breast cancer incidence and mortality than women with low SES.^[4,7,8]^ The absolute number of breast cancers prevented by mammographic screening may, therefore, be higher in women with high SES than in women with low SES. The prerequisite is that the effectiveness of mammographic screening in women with high SES is equal to or greater than the effectiveness of screening in women with low SES.^[9]^ Because high SES is associated with a higher breast density,^[10]^ which can mask tumors on mammograms^[11]^; the relative effect of screening might actually be smaller in women with high SES than low SES.

Only 1 study^[9]^ investigated the relative effect of mammographic screening in younger women (aged 40–49 years) with either high or low SES. In women aged 40 to 49 years, the relative effect of mammographic screening on breast cancer mortality did not differ significantly between women with high and low SES.^[9]^ However, the effect of screening on breast cancer mortality in women with either high or low SES remains unknown in the most commonly targeted age groups.^[1]^ Therefore, the aim of this study is to investigate the relative effect of mammographic screening on breast cancer mortality in women with either high or low SES (based on income, employment, and education) aged 50 to 75, 40 to 75, and 50 to 69 years.

## 2 Methods

### 2.1 Setting

The case-control study was conducted within the population invited to the biennial mammographic screening program in Nijmegen, the Netherlands. In 1975, at the initiation of the program, women aged 35 years and over were invited for screening. In 1989, at the start of the national screening program, the targeted age range was limited to 50 to 69 years conforming to national screening policy. In 1998, the national program also started to invite women aged 70 to 75 years. Until 2014, the first screening examination consisted of 2 views (mediolateral oblique and craniocaudal view), and the subsequent screening examinations consisted of 1 view (lateral view in first 3 rounds and mediolateral view from the fourth round onwards). Additional craniocaudal views during subsequent screening increased over time. From 2014 onwards, 2 views became standard in subsequent screening examinations (mediolateral oblique and craniocaudal view). Mammograms are read independently by 2 radiologists who must reach consensus on recall. In 2007 to 2008, digital mammography was introduced.

The Nijmegen screening registry holds data on screening attendance, age, and postal code per screening round. It also collects information on vital status (date of death or migration) and cause of death of women diagnosed with breast cancer who are living in Nijmegen. Vital status is obtained from the Municipal Personal Records Database (GBA), and cause of death is assessed by a committee that is unaware of the screening history. All women consented to the use of their anonymous data for scientific research.

### 2.2 SES indicator

Socioeconomic status was based on the scores of the Netherlands Institute for Social Research (SCP score).^[12]^ This SCP score is available for all 4-digit postal codes with more than 100 households and is provided every 4 years by the Netherlands Institute for Social Research since 1995. The score is based on mean household income, percentage of households with a low income, percentage of inhabitants without a paid job, and percentage of households with a low mean education. This information is obtained via phone calls from the organization Evers Direct Marketing Besloten Vennootschap (EDM-BV) to 1 person in each 6-digit postal code (usually 1 street) and aggregated to 4-digit postal codes.

We made groups of high and low SES based on the SCP score using the mean score of each 4-year period (−0.26 for 1995–1998, −0.12 for 1999–2002, 0.21 for 2003–2006, and 0.17 for 2007–2010). The SES indicator in the period 1995 to 2002 was used to determine the SES in the period 1975 to 1994, assuming that the SES did not change from high to low or vice versa in this time period. Nijmegen had a lower mean SES score than the Netherlands and covered about one-third of the total range of the SCP scores.

### 2.3 Study design

We used a case-control study to evaluate the effect of mammographic screening on breast cancer mortality in women with a high and low SES (see Supplement 1 for STROBE checklist). Cases were defined as women who were aged 50 to 75, 40 to 75, or 50 to 69 years at diagnosis, were invited to participate in the mammographic screening program, were living in Nijmegen, and died from breast cancer before January 1, 2013. For each case, 5 controls were sampled according to the incidence density sampling procedure using the syntax of Richardson.^[13]^ Controls had to be of the same age range as the cases, invited to participate in the screening program at the time of diagnosis of the case, living in Nijmegen, and alive at the time of death of the case.

Breast cancer screening is only effective in the period that breast cancer is detectable by the screening test and not yet symptomatic. Because the detectable preclinical period is unknown at individual level, we set the time frame for invitation at 4 years before the diagnosis of the case based on estimated lead times for breast cancer.^[14,15]^ In biennial screening, a 4-year period covers 2 consecutive screening invitations: the invitation before breast cancer diagnosis of the case (index invitation) and the invitation preceding the index invitation (preindex invitation). The age at index invitation and SES at index invitation were used to determine the age and SES, respectively, of both the cases and referents.

### 2.4 Statistical analyses

**Figure 1:**
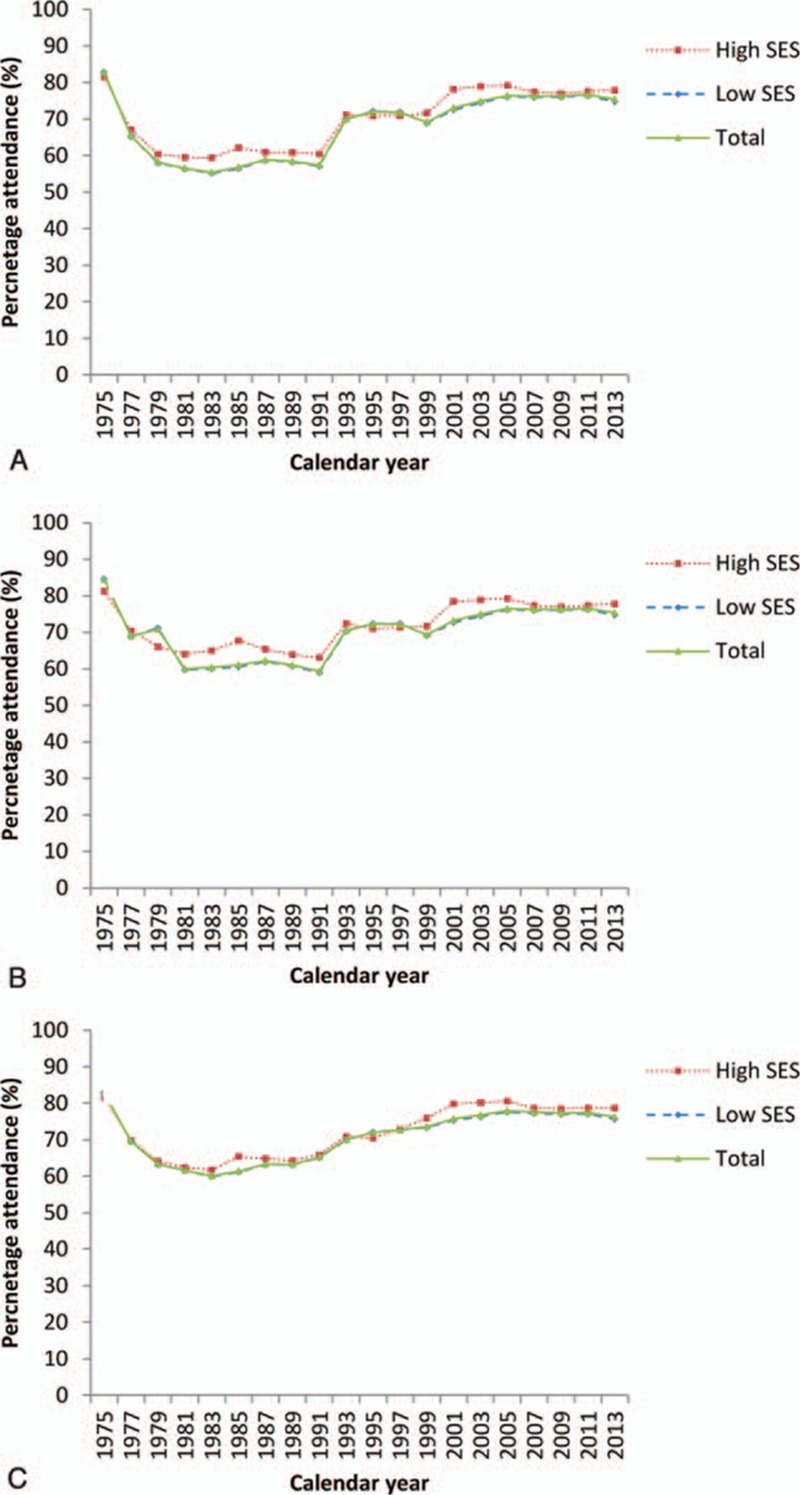
The screening attendance over time for women with a high and low SES aged 50 to 75 (A), 40 to 75 (B), and 50 to 69 (C) years. SES = socioeconomic status.

We used a chi-square test for independence to compare the clinical characteristics of the cases with high and low SES. Unconditional logistic regression was used to calculate the odds ratio (OR) of the breast cancer mortality rate in women with high and low SES accepting or declining the screening invitation. Unconditional logistic regression results in an unbiased OR if the proportion of women screened remains stable during the study period.^[16]^ In the Nijmegen screening program, the percentage of attendance was relatively stable (Fig. 1).

We calculated ORs and their corresponding 95% confidence intervals (95% CIs) for women with high and low SES separately and combined. The ORs were calculated for 3 age ranges, thereby covering most of the targeted age ranges in Europe: 50 to 75 (the Netherlands), 40 to 75 (Sweden), and 50 to 69 (Norway, Italy).^[1]^ All analyses were adjusted for age and executed in SAS version 9.2 (SAS Institute Inc., Cary, NC).

## 3 Results

### 3.1 Study population

In the period 1975 to 2012, 10% of the women invited for screening had a high SES and 89% of the women had a low SES. SES was unknown for 0.24% of the women aged 50 to 75 years. The percentage of high SES increased over time: from 4% in 1975 to 20% in 2012. The attendance percentages were slightly higher for women with high SES than for women with low SES (see Fig. 1A–C). For women aged 50 to 75 years, the average attendance was 67.4% for low SES and 73.0% for high SES. For women aged 40 to 75 and 50 to 69 years, the average attendance was 69.1% and 70.7% for low SES, and 73.1% and 72.3% for high SES, respectively.

### 3.2 Case-control study

In the study period, 370 women aged 50 to 75, 451 women aged 40 to 75, and 313 women aged 50 to 69 years died from breast cancer (cases). Table 1 presents the clinical characteristics of the cases stratified by SES, which shows that the cases with high and low SES do not differ statistically significantly with respect to mode of detection, age at diagnosis, lymph node status, tumor size, breast density, estrogen receptor (ER), and progesterone receptor (PR) status. The cases with high and low SES differed only significantly with respect to treatment, that is, type of surgery and therapy after surgery (see Table 1). We randomly sampled 5 controls per case, leading to 1850, 2255, and 1565 controls in the age groups 50 to 75, 40 to 75, and 50 to 69 years, respectively. The median age at index invitation was 61 (interquartile range [IQR] 55–67) for the cases aged 50 to 75 years, and 59 (IQR 54–66) for the controls aged 50 to 75 years. For women aged 40 to 75 and 50 to 69 years, the median age at index invitation was 58 years (IQR 51–66) and 59 years (IQR 54–63) for cases, and 57 years (IQR 50–64) and 58 years (IQR 54–63) for controls, respectively.

**Table 1:**
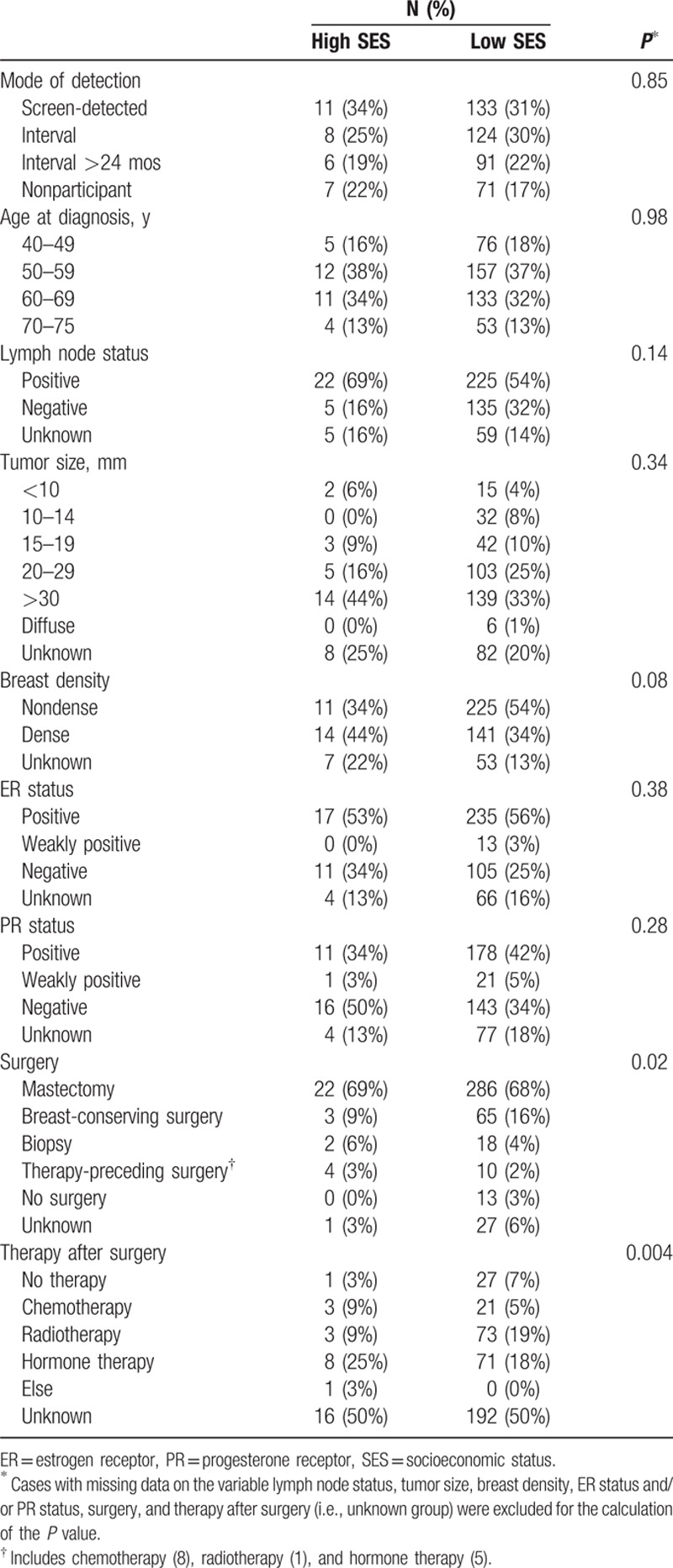
Clinical characteristics of cases aged 40 to 75 years at diagnosis.

Table 2 shows that screened women experience a lower breast cancer mortality rate than unscreened women. The overall mortality reduction by screening adjusted for age was 38% (OR 0.62, 95% CI 0.49–0.80) for women aged 50 to 75 years, 29% (OR 0.71, 95% CI 0.57–0.89) for women aged 40 to 75 years, and 38% (OR 0.62, 95% CI 0.48–0.82) for women aged 50 to 69 years. The effect of SES on the effectiveness of mammographic screening was not significant and differed with age group. The effectiveness was higher for high SES than for low SES in women aged 40 to 75 and 50 to 69 years, whereas the effectiveness was lower for high SES than for low SES in women aged 50 to 75 years. For women aged 50 to 75 years, the age-adjusted mortality reduction by screening was 39% (OR 0.61, 95% CI 0.47–0.78) for low SES and 18% (OR 0.82, 95% CI 0.31–2.19) for women with high SES. For women aged 40 to 75 years, the age-adjusted mortality reduction by screening was 28% (OR 0.72, 95% CI 0.57–0.91) for low SES and 43% (OR 0.56, 95% CI 0.25–1.29) for high SES.

**Table 2:**
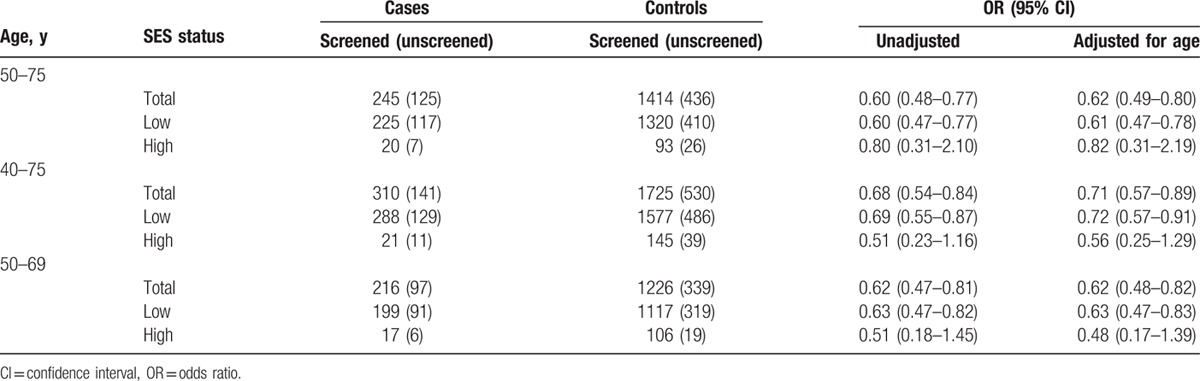
The relative effect of mammographic screening on breast cancer mortality for women with a high and low socioeconomic status unadjusted and adjusted for age.

A range of 2 to 10 women, selected either as case or referent, had a missing SES at the index round. The ORs changed maximal 0.03 point when women with missing SES at the index round were included in the analyses using the SES of another round with known SES and closest to the index round.

## 4 Discussion

To the best of our knowledge, this is the first study investigating the relative effect of screening on breast cancer mortality in women with high and low SES in the target age range of most European screening programs. We showed that mammographic screening reduces breast cancer mortality in women with high and low SES, but that the relative effectiveness of screening does not differ significantly between women with high SES and low SES in this study.

### 4.1 Effectiveness

So far, only 1 study investigated the effectiveness of mammographic screening for women with high and low SES aged 40 to 49 years.^[9]^ This study showed, like the study reported here, no statistically significant difference in the relative effectiveness of mammographic screening by SES. The absence of a difference in the relative effect of mammographic screening by SES may be real or not found because of limitations of the current study.

A difference in the relative effect of mammographic screening by SES may be expected because women with high and low SES may differ in treatment,^[17]^ mammography test characteristics,^[18]^ and/or breast cancer awareness.^[19]^ Adequate treatment after the detection of breast cancers by screening is essential for screening to be effective.^[20]^ Previous studies indicated that treatment inequalities between women with low SES and high SES may explain the lower survival from screen-detected breast cancer for women with low SES.^[17,21]^ In the current study, we found also a significant difference in type of surgery and therapy after surgery between cases with high and low SES. Women with low SES had more frequent no surgery (due to refusal of the patient, patient's age, or patient's medication) or no treatment after surgery than women with high SES. The main source of the treatment inequalities is probably not SES itself, but rather the higher presence of comorbid conditions in women with low SES.^[22,23]^ Based on the differences in treatment, it can be expected that the relative effect of mammographic screening is smaller for women with low SES than for women with high SES.

However, there are more factors that can potentially influence the effectiveness of mammographic screening by SES and that have an opposite effect, that is, mammographic test characteristics and breast cancer awareness. Mammographic screening test characteristics, such as sensitivity, may be lower for women with high SES. This is mainly because SES is positively associated with breast density,^[10]^ and high breast density can mask tumors on mammograms.^[11]^ SES itself is not likely to affect the screening test characteristics in the Netherlands, because quality of screening does not depend on SES and evaluating radiologists are unaware of SES. Furthermore, the relative effect of mammographic screening may be smaller for women with high SES than for women with low SES, because women with high SES have higher breast cancer awareness.^[19]^ A high breast cancer awareness is associated with cancer-related behavior such as healthcare seeking.^[19]^ As a consequence, women with high SES who did not participate in the index or the preindex round may still have been detected early, leading to a smaller effect of screening in women with high than low SES. Because the effect of treatment and breast cancer awareness are in an opposite direction, if existing in our study population, they may cancel out and result in an unobservable effect in this study.

### 4.2 Limitations and strengths

The absence of a difference in the relative effect of mammographic screening may also be the result of limitations of the current study. In this study, SES was based on an area-based measure, which does not capture individual as well as an individual-level SES measure.^[24]^ Furthermore, we extrapolated the SES of the period 1995 to 1998 to the period 1975 to 1994. These 2 limitations of the SES indicator used in this study may have caused misclassification of SES and attenuated the effect of SES towards the null. Besides this, Nijmegen had in general a low SES, especially in the early years, leading to a small number of cases with high SES. As a consequence, the OR for women with high SES had wide CIs. This problem can be overcome by using a larger number of cases, that is, by linking national cancer and screening registries. We would, however, like to point out that Hellquist et al^[9]^ also found no significant difference in the effectiveness of mammographic screening by SES, even though this study had an individual measure and a high number of women who died from breast cancer.

Our study also had strengths and limitations related to study design, study population, and external validity. Major strengths of our study were the use of a population-based approach, histological ascertainment of breast cancer, and accurate ascertainment of cause of death by a panel. We had a long period of follow-up, which has both advantages and disadvantages. The long follow-up of this study resulted from the nature of our study, that is, prospective and population-based, which is a strength. However, it should be recognized that during the follow-up period, the screening program had changed, for example, in the number of mammograms taken and in the radiological equipment. Another limitation of our study is the risk of self-selection bias, because we compared attenders with nonattenders who may have a different background risk of breast cancer. Self-selection bias in the Netherlands is, however, is small,^[25]^ and we did not adjust for self-selection because it is related to SES. Furthermore, our study population covered only about one-third of the SES range in the Netherlands, thereby excluding the most extreme SES groups. We cannot exclude the possibility that the highest and lowest SES groups have a different impact on the effectiveness of mammographic screening, although this seems unlikely. Caution should also be taken in generalizing the results of this study to other countries, because SES is not an uniform concept^[26]^ and depends on cultural factors.^[8]^ We did, however, use a combination of factors that are often use to conceptualize SES, that is, education, income, and employment, and this combination of factors may have the same effect on the effectiveness of mammographic screening in countries that are comparable with the Netherlands.

### 4.3 Usefulness of SES in personalized screening

Finally, we would like to discuss the potential usefulness of SES in personalized screening. Factors relevant for personalized screening should be able to differentiate women with a more favorable benefit–harms balance from women with a less favorable, or even unfavorable, benefit–harm balance. SES seems to have this potential: assuming that the effectiveness of screening does not vary by SES, it can be expected that the absolute number of breast cancer deaths prevented by screening, that is, the benefit, is higher for women with a high SES. However, the harms of screening (false-positives, false-negatives, and overdiagnosis) should not be disproportionally higher for women with high SES than for women with low SES. We would further like to point out that discussion is needed to decide whether it is ethical to use SES as a factor for personalized screening. It is well-known that women with high SES generally have lower all-cause mortality and higher life expectancy. Thus, if a personalized screening program would result in a more favorable benefit–harm balance for women with high SES than low SES, this would lead to further health inequality.

## 5 Conclusions

To conclude, mammographic screening reduces breast cancer mortality in women targeted in most European mammographic screening programs, that is, women aged 50 to 75, 40 to 75, and 50 to 69 years. We did not observe a difference in the relative effect of mammographic screening on the breast cancer mortality between women with high and low SES. If the absence of a difference in the effectiveness of mammographic screening in women with low and high SES is real, the absolute number of breast cancers prevented will be higher for women with high SES than for women with low SES.

## Acknowledgments

We want to thank “Bevolkingsonderzoek Oost” for the provision of data on screening invitation and attendance of each woman living in Nijmegen since 1990.

## Supplementary Material

Supplemental Digital Content
